# Association between ambient temperature and risk of stroke morbidity and mortality: A systematic review and meta‐analysis

**DOI:** 10.1002/brb3.3078

**Published:** 2023-06-02

**Authors:** Jing Wen, Li Zou, Ziwen Jiang, Yufeng Li, Jiaxin Tao, Yifang Liu, Wenning Fu, Xue Bai, Jing Mao

**Affiliations:** ^1^ School of nursing, Tongji Medical College Huazhong University of Science and Technology Wuhan China; ^2^ Department of Neurology Zhongnan Hospital of Wuhan University Wuhan China; ^3^ Department of Hand and Foot Micro‐Orthopaedics Taihe Hospital of Hubei Medical College Shiyan China; ^4^ Graduate School Huazhong University of Science and Technology Wuhan China; ^5^ Key Laboratory of Emergency and Trauma, Ministry of Education, College of Emergency and Trauma Hainan Medical University Haikou China

**Keywords:** meta‐analysis, stroke, systematic review, temperature

## Abstract

**Background:**

Previous studies have suggested that ambient temperature is associated with the morbidity and mortality of stroke although results among these investigations remained unclear. Therefore, the purpose of present meta‐analysis was to summarize the evidence of the relationship between ambient temperature and stroke morbidity and mortality.

**Methods:**

A systematic search of the PubMed, Embase, and Web of Science databases was from inception to April 13, 2022. The pooled estimates for heat ambient temperature and cold ambient temperature, which were defined as comparison between extreme hot or cold conditions and the reference or threshold temperature, were calculated utilizing a random‐effects model. A total of 20 studies were included in the meta‐analysis.

**Results:**

The pooled estimated show that the heat ambient temperature was significant associated with 10% (relative risk [*RR*], 1.10; 95% confidence interval [95%*CI*]: 1.02–1.18) and 9% (*RR*, 1.09; 95%*CI*: 1.02–1.17) increase in the risk of stroke morbidity and mortality, respectively. In addition, the pooled estimated show that the cold ambient temperature was significant associated with 33% (*RR*, 1.33; 95%*CI*: 1.17–1.51) and 18% (*RR*, 1.18; 95%*CI*: 1.06–1.31) increase in the risk of stroke morbidity and mortality, respectively.

**Conclusion:**

Integrated epidemiological evidence supports the hypothesis that both heat and cold ambient temperature have positive association with the risk of stroke morbidity and mortality. Targeted measures should be promoted in public health to reduce this risk.

## Graphical abstract

We conducted a meta‐analysis aim to summarize the evidence of the relationship between ambient temperature and stroke morbidity and mortality.For heat ambient temperature effect, the risk of stroke morbidity and mortality increased 10% (RR, 1.10; 95% CI: 1.02‐1.18) and 9% (RR, 1.09; 95% CI: 1.02‐1.17), respectively.For cold ambient temperature effect, the risk of stroke morbidity and mortality increased 33% (RR, 1.33; 95% CI: 1.17‐1.51) and 18% (RR, 1.18; 95% CI: 1.06‐1.31), respectively.

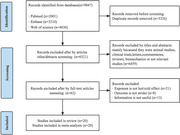

## 1 INTRODUCTION

Stroke is characterized by brain tissue damage due to a blockage of a blood vessel or a sudden rupture of a blood vessel, including ischemic stroke and hemorrhagic stroke, with high morbidity, mortality, and disability rates (Collaborators GCoD, 2018; Collaborators GDaIIaP, 2018). Stroke is currently still the second leading cause of death worldwide according to the World Health Organization 2020 report (Rana et al., 2021). Stroke not only is a public health issue but also contributes to a massive economic burden (Katan & Luft, 2018). Up till the present moment, there were approximately 34% of the global total health care expenditure spent on stroke. The economic burden of stroke per person in terms of cost of illness method was approximately equal to USD 1809.51‐325,108.84, including hospitalization, rehabilitation exercises, and follow‐up long‐term care (Rochmah et al., 2021). With the growing problems of population aging around the world, the global health care and economic burden of stroke are expected to continue to multiply in the coming years (Feigin et al., 2014). Moreover, previous studies from the Global Burden of Disease Study showed that approximately 90% burden of stroke could be attributed to lifestyle and environmental risk factors (Danaei et al., 2011; Feigin et al., 2016; Finucane et al., 2011). Therefore, we need a clearer comprehension of the various pathways from these risk factors to stroke, and every measurement must be taken to minimize the outcomes of stroke.

In the context of global climate change, the increased frequency, intensity, and duration of extreme temperature due to changes in ambient temperature and global warming have emerged as a public health threat, especially for populations with scant physiological ability or socioeconomic status to cope with or adapt to these changes. It contributes to a burden of disease and is set to move up in the coming years (Collaborators et al., 2018; McMichael, 2013). At the moment, the factors that trigger stroke have become the focus of attention and discussion in recent years to cope with changes that pose a fundamental threat to human well‐being and health, with published studies supported an association between extreme ambient temperature and an increased risk of stroke morbidity or mortality (Roye et al., 2019; Wang & Lin, 2014).

Although several studies have investigated the relationship between ambient temperature and the risk of stroke, the evidence has been conflicting (Breitner et al., 2014; Guo et al., 2017; Hong et al., 2003; Ikefuti et al., 2018; Wang & Lin, 2014). Although previous meta‐analysis explored the pooled relative risk (*RR*) of stroke for temperature changes, there were some limitations in the results (Wang et al., 2016). First, this studies only reported the relationship among daily mean temperature, daily maximum and minimum temperature, and stroke with only eight studies included but did not investigate the relationship between specific ambient temperature changes and stroke, such as above or below a threshold temperature or reference temperature. Equally important, they did not separately report the effect of temperature changes on the stroke morbidity and mortality, due to a limited number of original studies were included. In addition, there were many new studies published, but the results have been reported inconclusive (Schulte et al., 2021; Zhan et al., 2022).

Considering the results on the influence of ambient temperature upon stroke have been inconsistent, we update those studies with a focus on ambient temperature connection with and stroke by pooling evidence from relevant epidemiological studies. In view of the increasingly serious climate change and the heavy burden caused by stroke, the aim of present study is to provide valuable information for primary prevention of stroke and reduction of global health care expenditure.

## 2 METHODS

The present meta‐analysis was conducted according to the Preferred Reporting Items for Systematic Reviews and Meta‐Analyses (PRISMA) statement and the Meta‐Analysis of Observational Studies in Epidemiology Group (MOOSE) guidelines (Page et al., 2021; Stroup et al., 2000).

### 2.1 Search strategy

Literature searches were conducted in the PubMed, Embase, Web of Science databases. We conducted a literature search from the establishment of the database to April 13, 2022, and limited to studies on humans, published in English, with the following keywords: “temperature” or “heat effect” or “cold effect” or “hot exposure” or “cold exposure” or “extreme weather” or “climate” and “ischemic stroke” or “hemorrhagic stroke” or “cerebrovascular accident” or “cerebrovascular disorders.” The detailed search strategy terms are provided in Table S1.

### 2.2 Eligibility criteria

In present meta‐analysis, we used the following inclusion criteria that met the PECOS approach: (1) population: total population; (2) exposure: heat ambient temperature was defined as extreme heat condition, such as 95th percentile or 90th percentile temperature conditions, and so on; cold ambient temperature was defined as extreme cold condition, such as 5th percentile or 10th percentile temperature conditions, and so on; (3) comparator: exposure to the reference or threshold temperature; (4) outcome: confirmed cases or deaths cases of any type of stroke according to International Classification of Diseases codes; (5) study design: time‐series studies or case‐crossover studies. Animal‐based studies, intervention studies, toxicological studies, qualitative evaluations, letters, reviews, commentaries, or studies that only reported cardiovascular disease events but did not specifically access stroke or cerebrovascular disorders were excluded. Detailed inclusion criteria are shown in Tables 1 and 2.

**TABLE 1:** Eligibility criteria for this review

PECO framework	Criteria
Population	Total population including female, male, children, and adult
Exposure	Heat effect or cold effect of temperature
Comparator	Reference or threshold temperature
Outcome	Stroke morbidity or mortality
Studies design	Time‐series studies or case‐crossover studies

**TABLE 2:** Characteristics of studies included in the review

Author, year	Period	Country	Study design	Sample size/number of cases	Exposure	Temperature range (°C)	Outcome	Model	Lag (day)	Controlled variables	Effect indicator	Population	NOS score
Xu et al. (2021)	2005–2013	Brisbane, Australia	Case‐crossover study	11,469	Mean temperature	/	Hospitalization of stroke (ICD‐10: 160–169)	Conditional logistic regression	0–7	Relative humidity, NO_2_, PM_10_	*OR*	Total population	8
Shan et al. (2021)	1993–2012	Atlanta, USA	Time‐series study	67	Maximum temperature	/	Emergency department visit of ischemic stroke (ICD‐9: 433–437)	Logic regression	0–3	Season, day of week, holiday	*RR*	Total population	7
Roye et al. (2019)	2001–2013	Madrid, Spain	Time‐series study	106,036	Apparent temperature	−5.8 to 33.2	Hospital admissions of ischemic stroke (ICD‐9: 433–435) Mortality of ischemic stroke (ICD‐10: 163–164)	Distributed lag nonlinear model (DLNM)	0–14	Relative humidity, PM, NO, O, SO, long‐term‐trend, seasonality	*RR*	>15 years old	9
Bai et al. (2018)	1996–2013	Ontario, Canada	Time‐series study	355,837	Mean temperature	−33.1 to 32.2	Hospitalizations of stroke (ICD‐10: 160–168)	Distributed lag nonlinear model (DLNM)	0–21	Relative humidity, NO_2,_ O_3_, a day‐of‐week indicator, holiday status, daily influenza visits, seasonality, and long‐term trends	*RR*	Total population	8
Luo et al. (2018)	2013–2014	Beijing, China	Time‐series study	147,624	Mean temperature	−12.9 to 30.1	Hospital admissions of ischemic stroke (ICD‐10: 163) and hemorrhagic stroke (ICD‐10: 160–161)	Distributed lag nonlinear model (DLNM)	0–21	Relative humidity, PM_2.5_, day of week (DOW) and holiday, long term, and seasonality	*RR*	Total population	8
Guo et al. (2017)	2013–2015	Guangzhou, China	Time‐series study	104,432	Ambient temperature	0.6–37.6	Hospital admissions of intracerebral hemorrhagic (ICD‐10: 161)	Distributed lag nonlinear model (DLNM)	0–21	Day of week, public holidays, long‐term and seasonal trends of daily strokes, air pollution, relative humidity, meteorological variables	*RR*	Total population	8
Chen et al. (2017)	2008–2015	Nanchang, China	Case‐crossover study	16,264	Ambient temperature	−15.2 to 40.9	Hospital admission of ischemic stroke (ICD‐10: 163) and hemorrhagic stroke (ICD‐10: 160–161)	Conditional logistic regression models	1–3	Confounder by oneself	*OR*	Total population	7
Goggins et al. (2012)	1999–2006	Hong Kong, China	Time‐series study	130,962	Mean temperature	8.2–31.8	Hospitalization of ischemic stroke and hemorrhagic stroke	Poisson generalized additive models	0–13	Day of the week and holiday effects, NO_2_, SO_2_, RSP, O_3_	*RR*	Total population	8
Yao et al. (2020)	2005–2019	Rizhao, China	Case‐crossover study	1682	Mean temperature	−11.4 to 33.1	Hospital admission of subarachnoid hemorrhage (ICD‐10: I60.0–I60.7)	Distributed lag nonlinear model (DLNM)	0–4	Day of the week, seasonality, and long‐term trend	*OR*	Total population	7
Hong et al. (2003)	1998–2000	Incheon, Korea	Case‐crossover study	545	Ambient temperature	−6.8 to 28.1	Hospital admission of ischemic stroke	Conditional logistic regression	0–1	Relative humidity, barometric pressure	*OR*	Total population	7
Chen et al. (2013)	1996–2008	Shenyang, Beijing, Tangshan, Taiyuan, Shanghai, Guangzhou, Hong Kong, China	Time‐series study	127,750	Mean temperature	−22 to 33.8	Mortality of stroke (ICD‐10: I60–I69)	Distributed lag nonlinear model (DLNM)	0–14	Long‐term and seasonal trends, day of the week, air pollution (PM_10_, SO_2_, NO_2_), relative humidity	*RR*	Total population	7
Breitner et al. (2014)	1990–2006	Bavaria, Germany	Time‐series study	/	Mean temperature	−15.3 to 29.2	Mortality of cerebrovascular diseases (ICD‐9: 430–438 or ICD‐10: I60–I69)	Distributed lag nonlinear model (DLNM)	0–14	Long‐term trend/seasonality, weekday variations, influenza epidemics, relative humidity, barometric pressure	*RR*	Total population	8
Schulte et al. (2021)	1998–2016	Switzerland	Time‐series study	63,367	Maximum temperature	3–40	Mortality and emergency hospital admissions of ischemic stroke (ICD10: I63) and hemorrhagic stroke (ICD: I60–I62)	Distributed lag nonlinear model (DLNM)	0–10	Year, month, weekday, region	*RR*	Total population	9
Ban et al. (2017)	2013–2015	43 counties, China	Time‐series study	531,794	Mean temperature	7.40–29.00	Mortality of stroke (ICD‐10: I60–I64)	Distributed lag nonlinear model (DLNM)	0–2	Daily PM_2.5_, 24 h‐average O_3_, holiday	*RR*	Total population	8
Wang et al. (2015)	2007–2009	Beijing, Shanghai, China	Time‐series study	/	Mean temperature	−9.4 to 34.6	Mortality of cerebrovascular diseases (ICD‐10: I60–I69)	Distributed lag nonlinear model (DLNM)	0–27	Relative humidity, wind speed, PM_10_, SO_2_, NO_2_, day of the week	*RR*	Total population	8
Fu et al. (2018)	2001–2013	India	Time‐series study	19,753	Mean temperature	22.79–26.46	Mortality of stroke (ICD‐10: I60–I67, I69)	Distributed lag nonlinear model (DLNM)	0–21	Day of week, seasonality, long‐term trend	*OR*	Total population	7
Ikefuti et al. (2018)	2002–2011	São Paulo, Brazil	Time‐series study	55,633	Mean temperature	15–25	Mortality of stroke (ICD‐10: I60–I69)	Distributed lag nonlinear model (DLNM)	0–5	Seasonality, pollutants, humidity, and days of the week	*RR*	Total population	8
Polcaro‐Pichet et al. (2019)	1981–2015	Quebec, Canada	Case‐crossover study	13,201	Minimum temperature	/	Mortality of hemorrhagic (ICD‐9: 430–432, ICD‐10: I60–I62) and ischemic stroke (ICD‐9: 433–434, ICD‐10: I63)	Conditional logistic regression	/	Barometric pressure, relative humidity	*OR*	Total population	7
Zhan et al. (2022)	2010–2016	Fujian, China	Time‐series study	435,579	Apparent temperature	−2.0 to 33.8	Hospitalization of stroke (ICD‐10: I60–I64)	Distributed lag nonlinear model (DLNM)	0–28	PM_10_, Season, long‐term trend, day of week, public holidays	*RR*	Total population	9
Wang et al. (2014)	2000–2009	Taiper, China	Time‐series study	6962	Mean temperature	8–33	Emergency room visits of cerebrovascular diseases (ICD‐10: I60–I69)	Distributed lag nonlinear model (DLNM)	0–4	Relative humidity, season, long‐term trend, day of week, wind speed, holiday, influenza	*RR*	Total population	8

Abbreviations: ICD, international classification of diseases; NOS, Newcastle–Ottawa scale; *OR*, odds ratio; *RR*, relative risk.

### 2.3 Study selection

We screened and identified the studies which met the eligibility criteria in a step‐by‐step manner. First, we screened the title and abstract based on predefined eligibility criteria. Second, two authors (J.W. and Y.L.) independently assessed the full text before performing the meta‐analysis, the reference lists of relevant original articles and relevant review articles were also scrupulously checked to avoid missing possible original articles. Additionally, discrepancies were resolved by discussion with the third author (J.M.).

### 2.4 Data extraction

Eligible articles were extracted independently in duplicate, and all reconciled by two authors (J.W. and Y.L.). Data were extracted on the following study characteristics: the last name of the first author, publication year, study period, country, study design, sample size, exposure, temperature range, outcome, model, lag days, population, effect indicator (hazard ratio, *RR*, or odds ratios [*OR*] with 95% confidence interval [*CI*]), and controlled variables (i.e., humidity, air pollution, holiday, day of week and season). Any differences in data extraction were resolved and confirmed by discussion with third authors (J.M.).

### 2.5 Quality assessment

The Newcastle–Ottawa scale (NOS) was used to evaluate the quality of time‐series and case‐crossover studies, respectively. The scale score was calculated based on the three sections including study group selection (4 points), comparability of groups (2 points), and the exposures or outcomes (3 points) (Higgins et al., 2003). Quality was graded as low (0–3 points), moderate (4–6 points), and high (7–9 points), with higher points indicating higher quality.

### 2.6 Data analysis

We performed a meta‐analysis to pool the estimates of *RR* from all included original epidemiological studies to measure the association between heat or cold ambient temperature effect and risk of stroke. As the absolute risk of stroke is less than 10%, the reported *OR* was considered approximately as *RR* (Egger et al., 1997). Since the heterogeneity from the study design, different methods of ambient temperature measure, different temperature threshold or reference, geographical location, and lag pattern between original studies, the pooled estimates were evaluated using the random‐effects model when the index of heterogeneity (*I* ^2^) was >25%; or otherwise, the fixed‐effects model was applied (Begg & Mazumdar, 1994). We calculated the pooled maximum estimate from each original study. When different separate risk estimates were given for different geographic locations in a study that did not provide an overall risk estimate, we combined the results of these estimates to calculate the overall risk estimates for meta‐analysis.

Heterogeneity across each study was valued by the *Q* statistic quantified and the *I* ^2^ statistic. In present study, statistical heterogeneity was expressed by a significant *Q* statistic at the *p* < .10. In addition, *I* ^2^ values were used to describe the degree of heterogeneity, with 25%, 50%, and 75% indicating low, moderate, and high heterogeneity, respectively (Higgins et al., 2003).

We performed subgroup analysis, sensitivity analysis, and publication bias assessment to investigate the possible heterogeneity and robustness of our meta‐analysis. We conducted subgroup analyses according to the potential influence of some factors, such as study design, socioeconomic status, latitude, and whether the studies controlled for relative humidity, air pollution, day of week, holiday, and long‐term and seasonal trend. The impact of individual studies on the pooled estimates was confirmed by conducting a sensitivity analysis using the leave‐one‐out method. Additionally, Egger's regression test, Begg's funnel plot, and the trim‐and‐fill method were used to test potential publication bias (Begg & Mazumdar, 1994; Duval & Tweedie, 2000; Egger et al., 1997).

All statistical analyses were conducted using Stata 16.0 (Stata Corporation, College Station, TX, USA), and *p* < .05 (two‐sided) was deemed statistically significance.

## 3 RESULTS

### 3.1 Study selection

**FIGURE 1:**
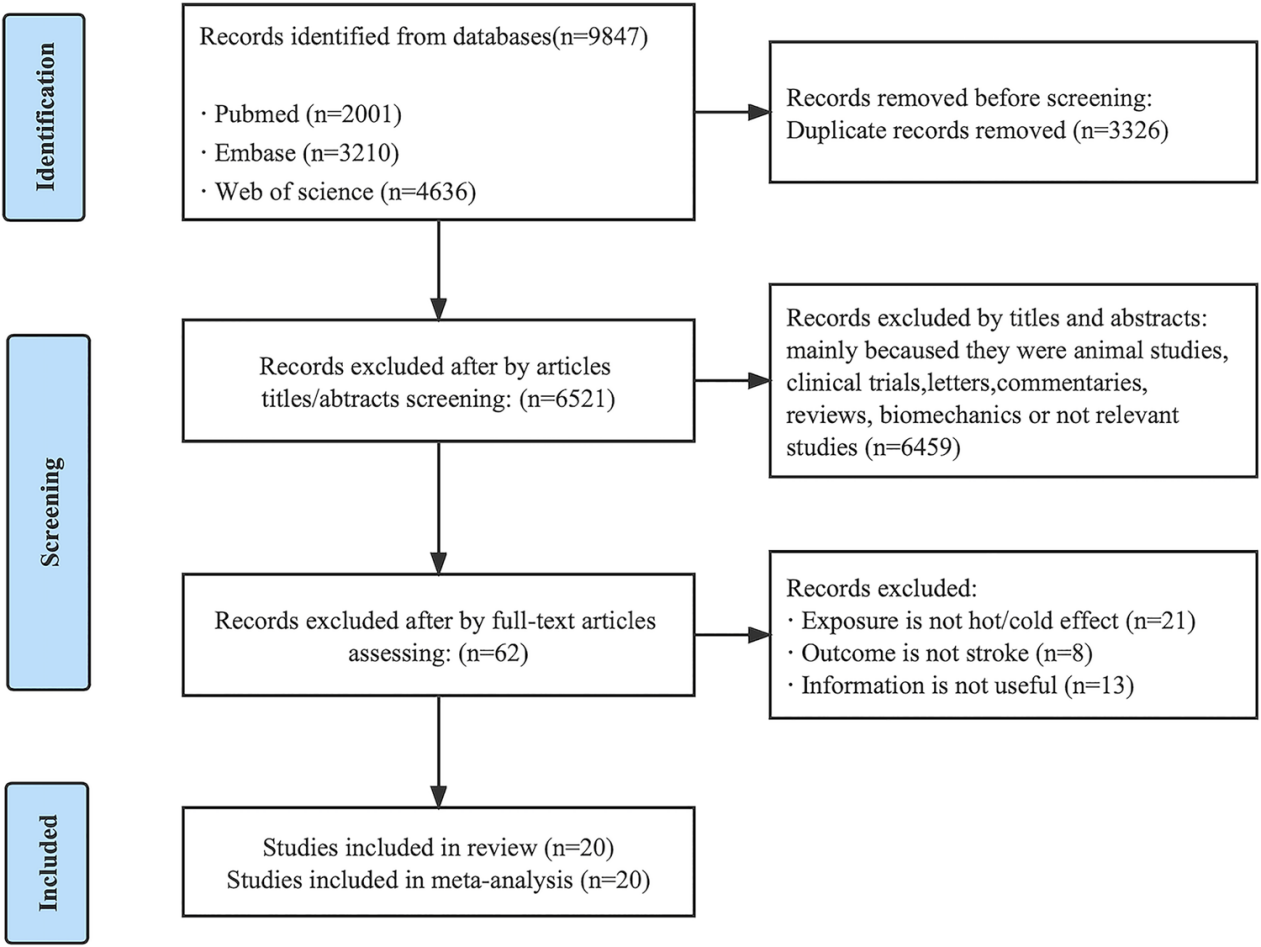
Flowchart of process for included studies into the meta‐analysis.

According to the systematic search, a total of 9847 articles were identified, of which 3326 were removed because of duplication. Of those, 6459 were removed after review of the title and abstract, because they were animal studies, clinical trials, letters, commentaries, reviews, biomechanics, or not relevant studies. Then, a total of 62 articles were excluded after a full‐text screening because exposure was not heat/cold effect, the outcomes were not stroke, or information were not useful. Finally, 20 articles were leaved on meta‐analysis (Bai et al., 2018; Ban et al., 2017; Breitner et al., 2014; Chen et al., 2013, 2017; Fu et al., 2018; Goggins et al., 2012; Guo et al., 2017; Hong et al., 2003; Ikefuti et al., 2018; Jiang et al., 2021; Luo et al., 2018; Polcaro‐Pichet et al., 2019; Roye et al., 2019; Schulte et al., 2021; Wang & Lin, 2014; Wang et al., 2015; Xu et al., 2021; Yao et al., 2020; Zhan et al., 2022). The PRISMA flowchart is shown in Figure 1.

### 3.2 Study descriptions

A total of 20 articles were included, and 12 included studies were conducted in Asian, 6 included studies were conducted in Europe and North American, and only one was conducted in South American and Australia, separately. Of these, 15 used a time‐series study design, and 5 used a case‐crossover study design, which published between 2003 and 2022. In addition, the average quality scores of time‐series studies were 8, ranged from 7 to 9, and the average quality scores of case‐crossover studies were 7.2, ranged from 7 to 8. In terms of included temperature exposure type and health outcome, 10 reported the heat ambient temperature effect of stroke morbidity, 11 reported the cold ambient temperature effect of stroke morbidity, 7 reported the heat ambient temperature effect of stroke mortality, and 7 reported the cold ambient temperature effect of stroke mortality. The daily mean temperature and daily maximum and minimum temperature were most commonly used as measures in the included studies, although some studies used apparent temperature. Temperature for all included studies ranged from −33.1 to 40.9°C. The lag effects of temperature were deemed among most studies ranged from 0 to 28 days, as heat ambient temperature exposures were immediate effect, and cold ambient temperature exposures were delayed effect. The most common method used to examine the relationship between ambient temperature and stroke morbidity or mortality was time‐series using distributed lag nonlinear models, whereas some studies were case‐crossover design using conditional logistic regression models.

### 3.3 Ambient temperature and risk of stroke morbidity and mortality

**FIGURE 2:**
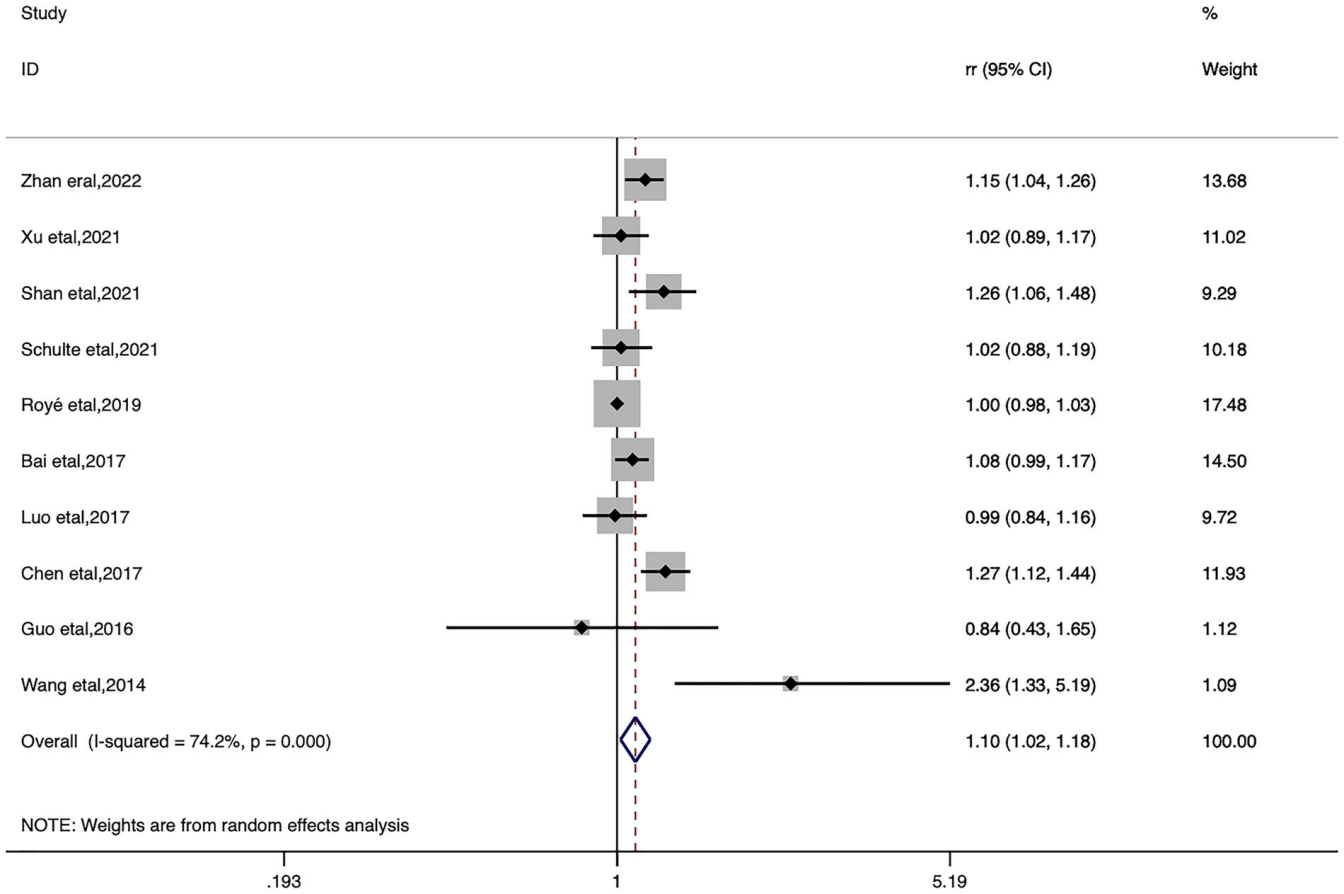
Forest plot of stroke morbidity for the heat ambient temperature.

**FIGURE 3:**
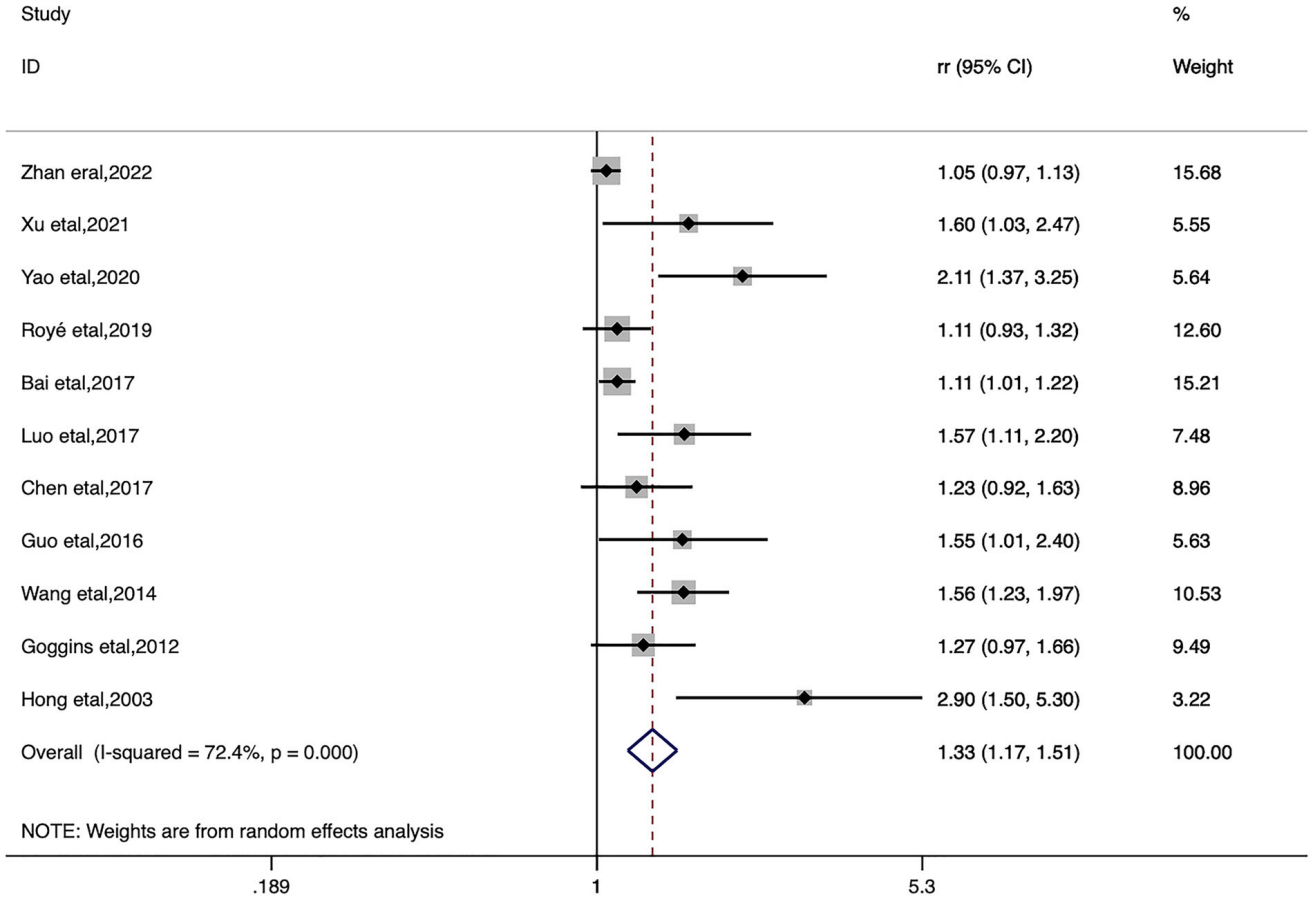
Forest plot of stroke morbidity for the cold ambient temperature.

**FIGURE 4:**
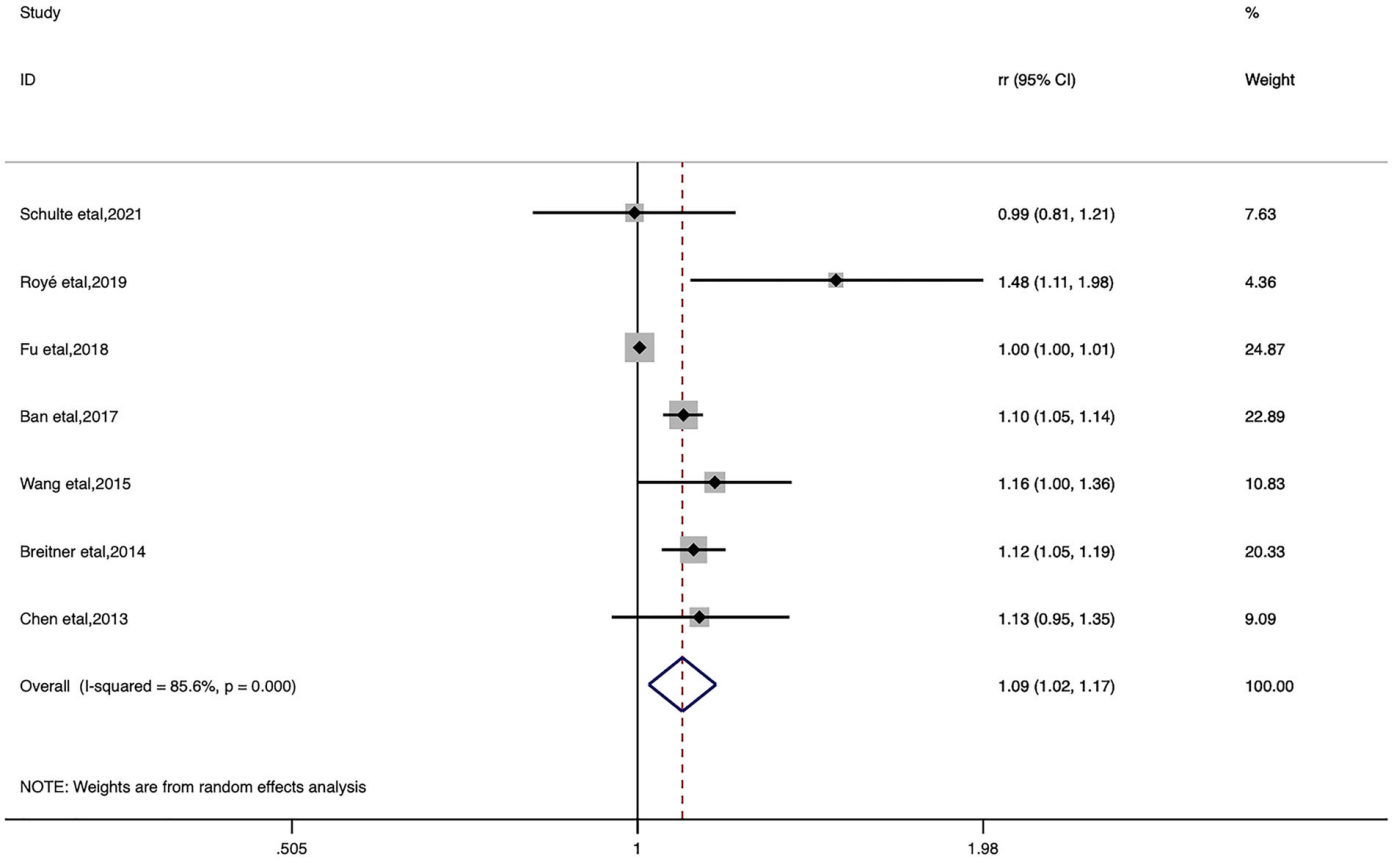
Forest plot of stroke mortality for the heat ambient temperature.

**FIGURE 5:**
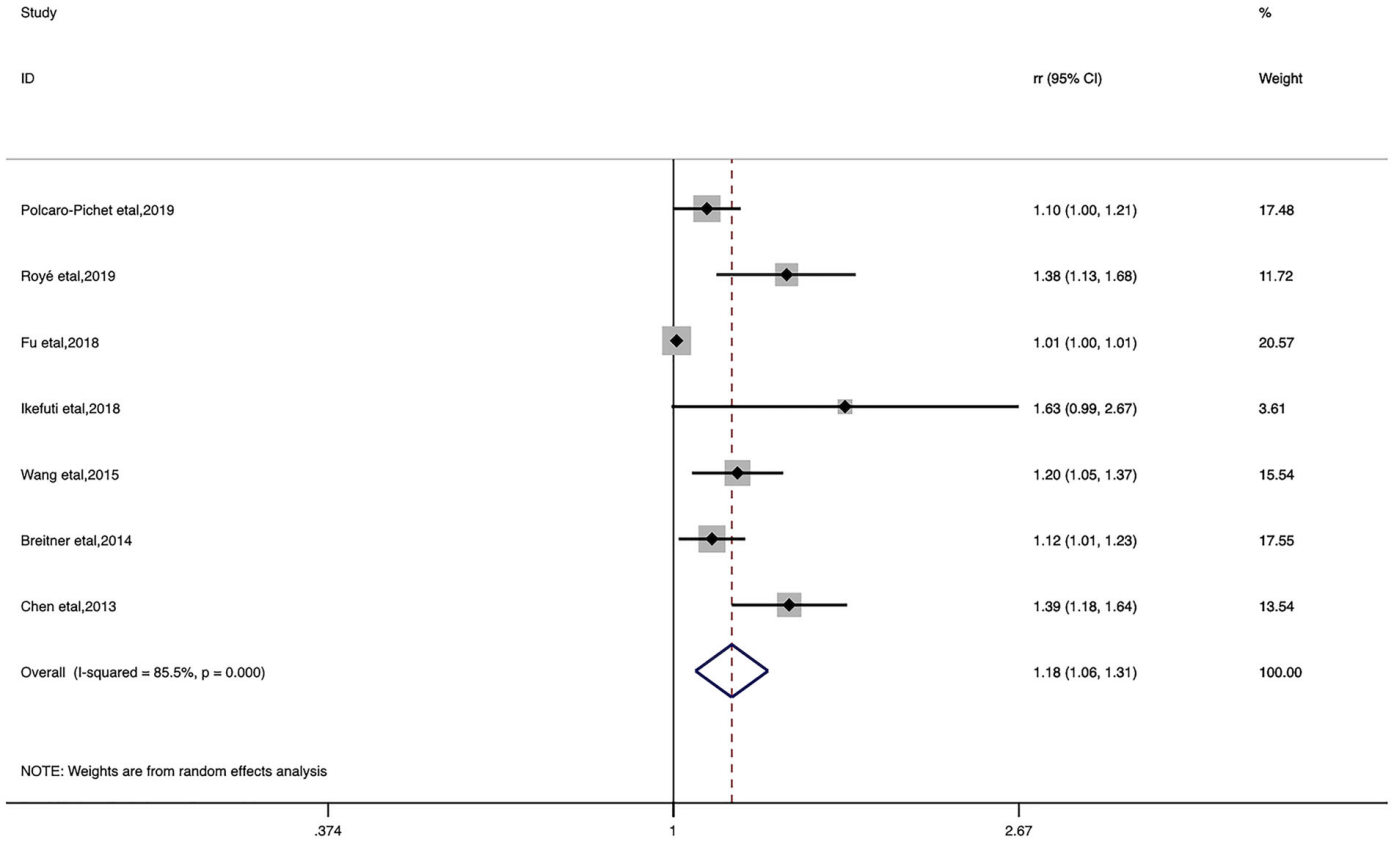
Forest plot of stroke mortality for the cold ambient temperature.

In the categorical meta‐analysis, the pooled estimates of heat ambient temperature effect on stroke morbidity and mortality and cold ambient temperature effect on stroke morbidity and mortality were shown separately (Figures 2, 3, 4, 5). We found that increase or decrease in temperature was positively correlated with the risk of morbidity and mortality from stroke compared to the reference or threshold value for temperature. For heat ambient temperature effect, the pooled *RR* of stroke morbidity was 1.10 (95%*CI*: 1.02–1.18; *I* ^2^ = 74.2%), and the pooled *RR* of stroke mortality was 1.09 (95%*CI*: 1.02–1.17; *I* ^2^ = 85.6%). For cold ambient temperature effect, the pooled *RR* of stroke morbidity was 1.33 (95%*CI*: 1.17–1.51; *I* ^2^ = 72.4%), and the pooled *RR* of stroke mortality was 1.18 (95%*CI*: 1.06–1.31; *I* ^2^ = 85.5%).

### 3.4 Subgroup analyses

Aim to evaluate the stability of the primary pooled estimates results and identify the possible resources of heterogeneity, subgroup analyses were conducted according to study design, socioeconomic status, latitude, and controlling for relative humidity, air pollution, day of week, holiday, long‐term, and seasonal trend, respectively. In general, the results were similar across the subgroups in that heat or cold ambient temperature increased the risk of stroke. When stratified by socioeconomic status, we found that stroke morbidity and mortality (*RR* = 1.16, 95%*CI*: 1.00–1.34; *RR* = 1.14, 95%*CI*: .97–1.33) from heat ambient effect were higher in developed countries compared to developing countries (*RR* = 1.05, 95%*CI*: .98–1.12; *RR* = 1.07, 95%*CI*: 1.00–1.16). Interestingly, the cold ambient effect was quite different, with stroke mortality and morbidity risks being lower in developed countries (*RR* = 1.13, 95%*CI*: 1.02–1.27; *RR* = 1.15, 95%*CI*: 1.04–1.28) than in developing countries (*RR* = 1.47, 95%*CI*: 1.19–1.81; *RR* = 1.22, 95%*CI*: 1.00–1.47). Of note, we found that the heterogeneity of the association between the heat ambient temperature effect and stroke mortality was 35.5% in studies that controlled for air pollution and 81.8% in studies that did not. In terms of cold ambient temperature effect and stroke mortality, the heterogeneity was 7.5% in studies that controlled for air pollution and 72.7% in studies that did not. Additionally, subgroup on the heat or cold ambient temperature effect of stroke mortality showed that the source of heterogeneity could also be whether the long‐term and seasonal trend was controlled. The heterogeneity was mainly from studies controlled for long‐term and seasonal trend: *I* ^2^ = 84.7% for heat ambient temperature effect and *I* ^2^ = 87.4% for cold ambient temperature effect. The heterogeneity was 0.0% and 10.0% for heat and cold ambient temperature effect that did not control the long‐term and seasonal trend, respectively. The result of the subgroup analysis is in Table S2.

### 3.5 Sensitivity analyses

Sensitivity analyses suggested that neither the study was likely to have a significant impact on the result for heat ambient temperature effect on stroke mortality, nor cold ambient temperature effect on stroke morbidity and mortality (Figure S1). For heat ambient temperature effect, the pooled *RR* ranged from 1.08 (1.01–1.15) to 1.11 (1.06–1.16) in stroke mortality. For cold ambient temperature effect, the pooled *RR* ranged from 1.28 (1.14–1.44) to 1.41 (1.20–1.66) and from 1.14 (1.04–1.26) to 1.21 (1.06–1.37) in stroke morbidity and mortality, respectively. Although sensitivity analysis suggested very subtle modifies in the assessments of heat ambient temperature effect on stroke morbidity, the pooled *RR* ranged from 1.07 (95%CI: 1.00–1.14) to 1.12 (95%CI: 1.03–1.20). The study performed by Chen would modify the pooled heat ambient temperature effect of stroke morbidity.

### 3.6 Publication bias

For heat ambient temperature effect on stroke morbidity and mortality, the Egger test indicated evidence of publication bias, but the Begg test did not (*p*_Egger's_ = .048, *p*_Begg's_ = .858 in stroke morbidity; *p*_Egger's_ = .021, *p*_Begg's_ = .764 in stroke mortality). For cold ambient temperature effect on stroke morbidity and mortality, both the Egger test and Begg test indicated evidence of publication bias (*p*_Egger's_ = .000, *p*_Begg's_ = .008 in stroke morbidity; *p*_Egger's_ = .001, *p*_Begg's_ = .023 in stroke mortality).

The pooled effects on stroke morbidity were corrected using the trim‐and‐fill method owing to publication bias. The corrected *RR* of heat ambient temperature effect on morbidity and mortality was 1.03 (95%CI: .95–1.11) and 1.04 (95%CI: .97–1.10), respectively. The corrected *RR* of cold ambient temperature effect on morbidity and mortality was 1.11 (95%CI: .97–1.27) and 1.03 (95%CI: .94–1.14), respectively. Funnel plots and trim‐and‐fill figures are in Figures S2 and S3.

## 4 DISCUSSION

This meta‐analysis supplies an updated examination of the effects of various ambient temperature conditions. To the best of our knowledge, this is the first study to assess not only the association between heat and cold ambient temperature effects and stroke morbidity, but also stroke mortality, which fill the gaps in previous studies. More specifically, present meta‐analysis found that the risk of stroke morbidity and mortality significantly increased in relation to heat and cold ambient temperature effect. Our findings have important implications for developing the policy of public health and reducing the burden of stroke.

The combined estimate of heat exposure on elevated risk of stroke morbidity and mortality, which is accordant with the previous meta‐analysis in which each 1°C increase was associated with 1.1% increase in occurrence and mortality of stroke (Lian et al., 2015). However, another meta‐analysis indicated no significant association between ambient maximum temperature and ischemic stroke occurrence (*RR*: .98, 95%*CI*: .94–1.02). The inconsistent results may be due to the fact that this meta‐analysis study reported the relationship between maximum temperature and ischemic stroke occurrence, which temperature as a continuous variable, but in our study ambient temperature as a categorical variable, heat ambient temperature was compared to reference or threshold temperature. Moreover, there were only two original studies with greater chance in previous meta‐analysis (Wang et al., 2016).

The increased risk of stroke morbidity and mortality with heat ambient temperature effect may be explained by some potential mechanisms. One possible explanation is that in heat conditions the body needs to increase heat diffusion through sweating, which may lead to dehydration and a reduction in central blood volume. This further leads to increased blood viscosity, altered serum osmolality, and altered BUN/cr, which may increase the risk of microvascular thrombosis and subsequent ischemic stroke (Bahouth et al., 2018; Donaldson et al., 2003; Keatinge et al., 1986; Qi et al., 2021). In addition, high temperatures may damage the endothelial cells of blood vessels and lead to coagulation dysfunction, and consequently to consumptive coagulation. This consumptive coagulation leads to excessive and prolonged multiple tissue hemorrhages resulting in multiple organ dysfunction syndrome, including cerebral hemorrhages (Lavados et al., 2018). At the same time, high temperatures can lead to a systemic inflammatory response due to a prolonged reduction in blood flow to the spleen and intestines. This would further lead to dysfunction of the coagulation system and hemodynamic disturbances, which is one of the possible explanations (Chaseling et al., 2021; Lavados et al., 2018; Leon & Helwig, 2010). There may be other mechanisms that require investigation as well.

In our meta‐analysis, the combined estimate of cold ambient temperature exposure on elevated risk of stroke morbidity and mortality was significant. Our assessment of the relationship between cold ambient temperature effect and stroke was in‐line with similar previous meta‐analyses (Lian et al., 2015). For example, the meta‐analysis showed that a 1°C decrease in temperature resulted in a 1.2% increase in stroke incidence and mortality. However, our results are inconsistent with the results of another meta‐analysis pooled effect size of only two original studies (Wang et al., 2016), possibly due to different types of temperature variables and chance from fewer original studies. Therefore, more epidemiology studies are required to provide more evidence on the association between ambient temperature and stroke morbidity or mortality.

Several physiological mechanisms by which cold ambient temperature effect might act as a trigger for stroke have been suggested to explain this phenomenon. Exposure to cold ambient temperatures may lead to activation of the sympathetic nervous system and the renin‐angiotensin system and an increase in blood pressure, which may lead to plaque rupture and thus an increased risk of ischemic stroke (Barnett et al., 2007; Ryti et al., 2017; Sun, 2010). In addition, cold‐induced vasoconstriction leads to reduced blood flow to the skin, whereas increased diuresis leads to concentrated and hyperviscous blood and increased fibrinogen and cholesterol levels, which are also a risk factor for the formation of blood clots (Keatinge et al., 1984; Liu et al., 2015; Neild et al., 1994). Moreover, hypothermia‐mediated changes in hematological properties can enhance forces favoring wall deformation and increase frictional and shear stresses on the inner surface of the vessel, which may increase the risk of hemorrhagic stroke (Vitale et al., 2015).

As substantial heterogeneity was found among the included studies, we conducted subgroup analyses by study design, socioeconomic status, latitude, and controlling for relative humidity, air pollution, day of week, holiday, long‐term and seasonal trend. In subgroup analyses, we found whether air pollution and long‐term and seasonal trends were controlled may be sources of heterogeneity. Besides, we found that heterogeneity remained substantial which may be related to numerous factors. First, exposure measurement of studies was from different sources with varied definitions. For example, some studies use apparent temperature, including daily average air temperature, relative humidity, wind speed, and vapor pressure, which is different from ambient temperature (Roye et al., 2019; Zhan et al., 2022). Moreover, there would also be differences between data from ground‐based weather stations and data from satellite space. Second, as heat ambient temperature effect and cold ambient temperature effect were defined the number of degrees above or below the threshold or reference value or a comparison between extreme ambient temperature conditions and the average value in this studies, different reference values or thresholds and varied definitions of extreme temperatures could be sources of heterogeneity. And this definition could introduce heterogeneity, because the risk function might be different in different temperature range. Moreover, the location of the studies would be expected to contribute to heterogeneity. In our subgroup analyses, we observed significant heterogeneity between the latitude subgroups on heat ambient temperature effect of stroke mortality. However, we cannot rule out chance in this result, and therefore, we should interpret this result with more caution. Owing to previous studies have mostly focused on low and middle latitudes, in the future we can conduct research in high latitudes to explore the effect of latitude on the stroke events. Other factors, including different study periods or socioeconomic conditions may also contribute to heterogeneity. Differences in socioeconomic status, such as between developed and developing countries, there may be differences in greenhouse gas emissions, in the prevalence of air conditioning, and in the responsiveness of health care systems in the face of extreme temperatures, all of which may be sources of heterogeneity. This supplies a direction for the further consideration of the heat or cold ambient temperature effect on stroke morbidity and mortality in terms of different socioeconomic conditions backgrounds.

Aim to access the robustness of our result, we conducted a publication bias assessment and a sensitivity analysis. Publication bias among studies which accessed the association between heat and cold ambient temperature effect and stroke morbidity and mortality were found in our study. Therefore, we used the trim‐and‐fill method to assess publication bias. We found that the trim‐and‐fill method could change our results, probably due to the small number of original studies. According to the principle of symmetry, multiple non‐existing small sample size studies were supplemented, and the combined effect size was calculated on this basis, resulting in the appearance of overcorrection (Egger & Smith, 1998). However, we verified that the association between heat and cold ambient temperature effect and stroke morbidity and mortality was positive and statistically significant by sensitivity analysis, even though the results of some studies were not statistically significant (Goggins et al., 2012; Guo et al., 2017; Luo et al., 2018; Schulte et al., 2021).

The review highlighted the need for research to identify and study interventions in public health to reduce the problem of increased stroke morbidity and mortality due to extreme ambient temperatures, particularly in vulnerable populations. First of all, all countries are advised to take environment‐related actions, such as reducing greenhouse gas emissions. Developed countries in particular should pay more attention to the public health issues arising from greenhouse gas emissions. The subgroup analysis showed that the risk of increased stroke morbidity and mortality associated with heat effect is higher in developed countries than in developing countries, which may be related to the higher greenhouse gas emissions in developed countries. Second, the health care system should also take action to deal with the health problems caused by extreme temperatures. For example, an early warning system for extreme temperatures should be set up, with extreme temperature warnings issued through television, radio, and the internet, so that the public, especially vulnerable people, can receive timely weather information and take measures to protect themselves. In addition, special adaptation measures and health promotion methods can also be developed for the characteristics of different vulnerable groups, which can lead to more effective results. For example, when extreme temperature warnings are issued, health care system such as community hospitals can take the initiative to give health guidance and medication advice to elderly people with chronic illnesses, especially those living alone and suffering from neurological disorders or a history of stroke, using telephone visits or home visits to ensure that they stay in air‐conditioned environments as much as possible during extreme temperature and seek prompt medical attention if they have any health problems. During a COVID‐19 pandemic, the health care system can take measures for smart care, such as telemedicine and smart homes, to reduce the health risks associated with COVID‐19‐related isolation.

There are several highlights in our study. First, we provided updates on the associations of ambient temperature with the risk of stroke, including more than one times as many studies as for a meta‐analysis of pooled effect sizes than previous meta‐analysis. Moreover, the selection of studies was based on strict criteria for eligibility and exclusion, which helped provide more reliable evidence. Second, in contrast to previous study, this is the first studies covered four outcomes, allowing comparisons among these outcomes and a more comprehensive assessment of ambient temperature changes effect on stroke morbidity and mortality separately. Third, despite heterogeneity among the included studies, consistent results from sensitivity analyses and various subgroup analyses suggest that our findings are reliable and robust.

However, in our studies there were some limitations should be of concern. First, we included both time‐series studies and case‐crossover studies. Although we used NOS for assessing the quality of included studies, which is considered the most effective standard. There are some limitations in proving causation of disease, which may influence the validity of our results (Mendoza & Yeh, 2021). Future multicenter, long‐term longitudinal studies are necessary to further investigate the effect of ambient temperature on the risk of stroke. Second, there was a high degree of heterogeneity between the included studies. Future investigations on a more precise temperature measurement standards or reference values are merited, and research in countries of different socioeconomic status would be worthwhile, particularly in developing countries.

## 5 CONCLUSIONS

The inspiration of our meta‐analysis clearly suggests that heat or cold ambient temperature are associated with an increased risk of stroke morbidity and mortality. This study provides a potential mechanism for the association of ambient temperature changes on stroke, and an understanding of the broader effects of extreme ambient temperature on stroke has the potential to suggest enhancements to public messaging efforts. As well as authorities should be aware of the seriousness and provide interventions that might mitigate the adverse effects of extreme ambient temperature, which everyone can benefit from these.

## AUTHOR CONTRIBUTIONS

**Jing Wen, Li Zou, and Jing Mao**: Conceptualization. **Jing Wen and Ziwen Jiang**: Methodology. **Jing Wen and Yufeng Li**: Software. **Jing Wen, Yufeng Li, and Li Zou**: Validation. **Ziwen Jiang, Wenning Fu, and Jiaxin Tao**: Formal analysis. **Ziwen Jiang, Wenning Fu, and Yufeng Li**: Investigation. **Ziwen Jiang, Wenning Fu, and Xue Bai**: Resources. **Xue Bai and Jiaxin Tao**: Data curation. **Jing Wen and Li Zou**: Writing—original draft preparation. **Yufeng Li and Li Zou**: Writing—review and editing. **Jing Mao**: Visualization. **Jing Mao**: Supervision. **Jing Mao**: Project administration. **Li Zou and Wenning Fu**: Funding acquisition

## CONFLICT OF INTEREST STATEMENT

The authors have no relevant financial or nonfinancial interests to disclose.

### PEER REVIEW

The peer review history for this article is available at https://publons.com/publon/10.1002/brb3.3078.

## Supporting information

Figure S1 Sensitivity analysis plot of stroke morbidity and mortality for ambient temperature.

Figure S2 Funnel plot of stroke morbidity and mortality for ambient temperature.

Figure S3 Filled funnel plot of stroke morbidity and mortality for ambient temperature.

Table S1 Search strategies for electronic academic databases.

Table S2 Subgroup analyses of stroke morbidity and mortality for ambient temperature.

## Data Availability

Some of the data and materials used in this study can be found in the Supporting Information section. More details can be obtained from the corresponding author (Jing Mao).
