# Supplementary figures and images for: Association between ambient temperature and risk of stroke morbidity and mortality: A systematic review and meta‐analysis
Source: Brain Behav. 2023 Jun 2;13(7):e3078. doi: 10.1002/brb3.3078 (PMC10338745; doi:10.1002/brb3.3078)

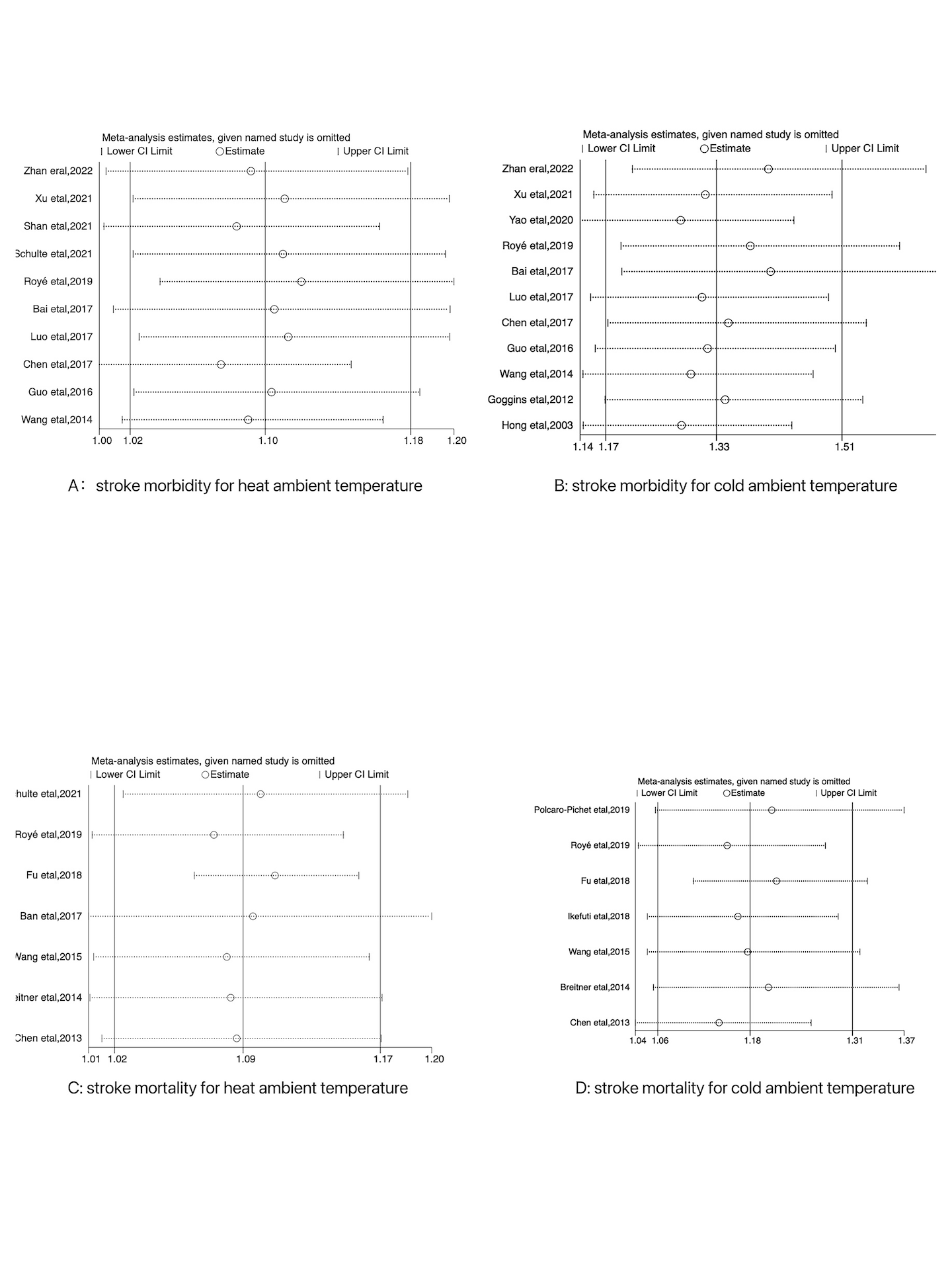

Supplement: Supplementary file 1 — Figure S1 Sensitivity analysis plot of stroke morbidity and mortality for ambient temperature. [file BRB3-13-e3078-s003.tif]

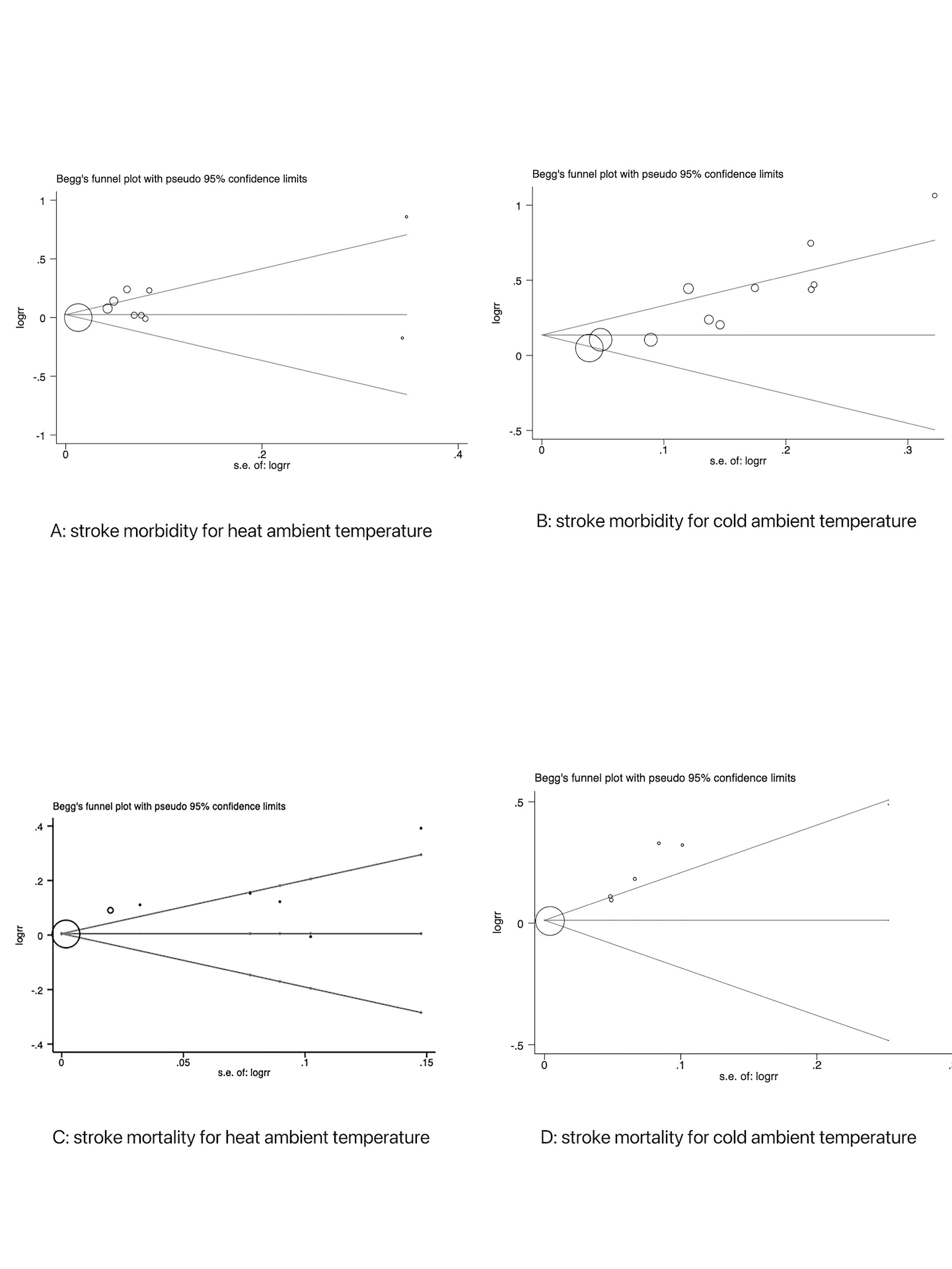

Supplement: Supplementary file 2 — Figure S2 Funnel plot of stroke morbidity and mortality for ambient temperature. [file BRB3-13-e3078-s002.tif]

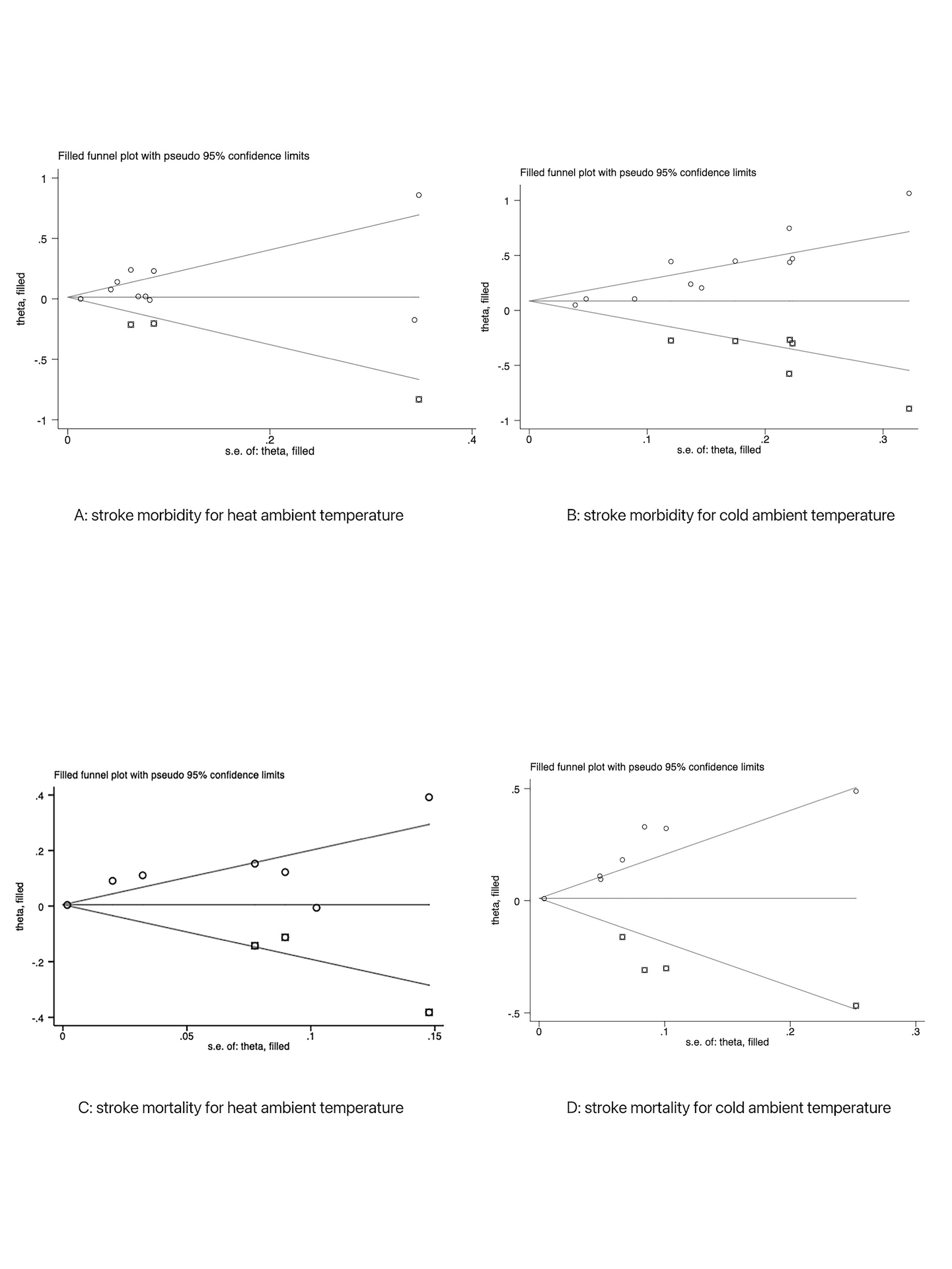

Supplement: Supplementary file 3 — Figure S3 Filled funnel plot of stroke morbidity and mortality for ambient temperature. [file BRB3-13-e3078-s005.tif]
