# Supplementary material for: Association between ambient temperature and risk of stroke morbidity and mortality: A systematic review and meta‐analysis
Source: Brain Behav. 2023 Jun 2;13(7):e3078. doi: 10.1002/brb3.3078 (PMC10338745; doi:10.1002/brb3.3078)
Supplement: Supplementary file 4 — Table S1 Search strategies for electronic academic databases. [file BRB3-13-e3078-s004.docx]

**Table S1 Search strategies for electronic academic databases**

| DATABASE/DATE | STRATEGIES |
| --- | --- |
| **Pubmed**  April 13, 2022 | 1. "Stroke"[MeSH Terms] OR "Hemorrhagic Stroke"[MeSH Terms] OR "Ischemic Stroke"[MeSH Terms] OR "Cerebrovascular Disorders"[MeSH Terms] 2. "cerebrovascular accident"[Title/Abstract] OR "cerebrovascular disorders"[Title/Abstract] OR "stroke"[Title/Abstract] OR "ischemic stroke"[Title/Abstract] OR "hemorrhagic stroke"[Title/Abstract] 3. 1 OR 2 4. "temperature"[MeSH Terms] OR "weather"[MeSH Terms] OR "extreme weather"[MeSH Terms] OR "atmosphere"[MeSH Terms] OR "climate"[MeSH Terms] OR "climate change"[MeSH Terms] 5. "hot exposure"[Title/Abstract] OR "cold exposure"[Title/Abstract] OR "heat effect"[Title/Abstract] OR "cold effect"[Title/Abstract] 6. 4 OR 5 7. 3 AND 6 8. Filters: English, Humans |
| **Embase**  April 13, 2022 | 1. (cerebrovascular accident or stroke or ischemic stroke or hemorrhagic stroke).ti. 2. (cerebrovascular accident or stroke or ischemic stroke or hemorrhagic stroke).ab. 3. (temperature or hot exposure or cold exposure or hot effect or cold effect).ti. 4. (temperature or hot exposure or cold exposure or hot effect or cold effect).ab. 5. 1 or 2 6. 3 or 4 7. 5 and 6 8. 7 and "human".sa_suba. |
| **Web of Science**  April 13, 2022 | 1. (((TS=(cerebrovascular accident)) OR TS=(stroke)) OR TS=(ischemic stroke)) OR TS=(hemorrhagic stroke) 2. ((((TS=(temperature)) OR TS=(hot exposure)) OR TS=(cold exposure)) OR TS=(hot effect)) OR TS=(cold effect) 3. 1 AND 2 4. 3AND English (Language) AND Humans (MeSH) |
