# Supplementary material for: Association between ambient temperature and risk of stroke morbidity and mortality: A systematic review and meta‐analysis
Source: Brain Behav. 2023 Jun 2;13(7):e3078. doi: 10.1002/brb3.3078 (PMC10338745; doi:10.1002/brb3.3078)
Supplement: Supplementary file 5 — Table S2 Subgroup analyses of stroke morbidity and mortality for ambient temperature. [file BRB3-13-e3078-s001.docx]

**Table S2 Subgroup analyses of stroke morbidity and mortality for ambient temperature**

| **Subgroup** | **Morbidity** | | | | | | | | **Mortality** | | | | | | | |
| --- | --- | --- | --- | --- | --- | --- | --- | --- | --- | --- | --- | --- | --- | --- | --- | --- |
|  | **Heat effect** | | | | **Cold effect** | | | | **Heat effect** | | | | **Cold effect** | | | |
|  | **No. of reports** | ***RR* (95% *CI*)** | ***P* for heterogeneity** | ***I^2^*(%)** | **No. of reports** | ***RR* (95% *CI*)** | ***P* for *heterogeneity*** | ***I^2^*(%)** | **No. of reports** | ***RR* (95% *CI*)** | ***P* for heterogeneity** | ***I^2^*(%)** | **No. of reports** | ***RR* (95% *CI*)** | ***P* for heterogeneity** | ***I^2^*(%)** |
| **All studies** | 10 | 1.10（1.02-1.18） | 0.000 | 74.2 | 11 | 1.33（1.17-1.51） | 0.000 | 72.4 | 7 | 1.09（1.02-1.17） | 0.000 | 85.6 | 7 | 1.18（1.06-1.31） | 0.000 | 85.5 |
| **Study design** |  |  |  |  |  |  |  |  |  |  |  |  |  |  |  |  |
| Time-series | 8 | 1.08（1.00-1.16） | 0.002 | 69.1 | 6 | 1.21（1.08-1.36） | 0.009 | 64.9 | 7 | 1.09（1.02-1.17） | 0.000 | 85.6 | 6 | 1.21（1.06-1.37） | 0.000 | 87.0 |
| Case-crossover | 2 | 1.14（0.92-1.42） | 0.019 | 81.9 | 4 | 1.75（1.23-2.50） | 0.040 | 63.8 | - | - | - | - | 1 | 1.10（1.00-1.21） | - | - |
| **Latitude** |  |  |  |  |  |  |  |  |  |  |  |  |  |  |  |  |
| Low | 4 | 1.22（1.05-1.42） | 0.091 | 53.7 | 5 | 1.27（1.06-1.54） | 0.008 | 70.9 | 1 | 1.00（1.00-1.01） | - | - | 2 | 1.20（0.76-1.89） | 0.057 | 72.3 |
| Middle | 6 | 1.04（0.98-1.10） | 0.077 | 49.6 | 6 | 1.46（1.17-1.83） | 0.001 | 76.5 | 6 | 1.11（1.07-1.16） | 0.323 | 14.3 | 5 | 1.20（1.10-1.31） | 0.049 | 58.1 |
| High | - | - | - | - | - | - | - | - | - | - | - | - | - | - | - | - |
| **Socioeconomic status** |  |  |  |  |  |  |  |  |  |  |  |  |  |  |  |  |
| Developed country | 5 | 1.16（1.00-1.34） | 0.043 | 59.4 | 3 | 1.13（1.02-1.27） | 0.274 | 22.8 | 3 | 1.14（0.97-1.33） | 0.085 | 59.4 | 3 | 1.15（1.04-1.28） | 0.119 | 53.0 |
| Developing country | 5 | 1.05（0.98-1.12） | 0.026 | 63.8 | 8 | 1.47（1.19-1.81） | 0.000 | 79.0 | 4 | 1.07（1.00-1.16） | 0.000 | 87.5 | 4 | 1.22（1.00-1.47） | 0.000 | 87.9 |
| **Control for relative humidity** |  |  |  |  |  |  |  |  |  |  |  |  |  |  |  |  |
| Yes | 7 | 1.05（0.98-1.13） | 0.012 | 63.2 | 8 | 1.30（1.13-1.49） | 0.000 | 74.5 | 4 | 1.15（1.07-1.23） | 0.309 | 16.5 | 6 | 1.21（1.11-1.33） | 0.047 | 55.6 |
| No | 3 | 1.18（1.03-1.36） | 0.062 | 64.1 | 3 | 1.42（1.07-1.89） | 0.094 | 57.7 | 3 | 1.04（0.96-1.12） | 0.000 | 89.2 | 1 | 1.01（1.00-1.01） | - | - |
| **Control for air pollution** |  |  |  |  |  |  |  |  |  |  |  |  |  |  |  |  |
| Yes | 6 | 1.04（0.99-1.11） | 0.065 | 51.9 | 6 | 1.16（1.04-1.29） | 0.056 | 53.7 | 4 | 1.14（1.05-1.24） | 0.199 | 35.5 | 4 | 1.31（1.19-1.44） | 0.356 | 7.5 |
| No | 4 | 1.22（1.04-1.44） | 0.024 | 68.2 | 5 | 1.57（1.24-1.99） | 0.039 | 60.3 | 3 | 1.04（0.96-1.14） | 0.004 | 81.8 | 3 | 1.06（0.98-1.14） | 0.026 | 72.7 |
| **Control for day of week** |  |  |  |  |  |  |  |  |  |  |  |  |  |  |  |  |
| Yes | 6 | 1.12（1.02-1.24） | 0.062 | 52.4 | 6 | 1.25（1.09-1.42） | 0.004 | 70.7 | 5 | 1.07（0.99-1.16） | 0.003 | 75.6 | 5 | 1.17（1.03-1.34） | 0.000 | 86.2 |
| No | 4 | 1.07（0.96-1.19） | 0.003 | 78.9 | 5 | 1.55（1.14-2.10） | 0.004 | 73.7 | 2 | 1.23（0.92-1.64） | 0.043 | 75.5 | 2 | 1.21（0.97-1.51） | 0.044 | 75.4 |
| **Control for holiday** |  |  |  |  |  |  |  |  |  |  |  |  |  |  |  |  |
| Yes | 6 | 1.12（1.02-1.24） | 0.062 | 52.4 | 7 | 1.32（1.13-1.53） | 0.000 | 76.2 | 1 | 1.10（1.05-1.14） | - | - | - | - | - | - |
| No | 4 | 1.07（0.96-1.19） | 0.003 | 78.9 | 4 | 1.42（1.05-1.93） | 0.020 | 69.4 | 6 | 1.10（1.01-1.20） | 0.000 | 78.5 | 7 | 1.18（1.06-1.31） | 0.000 | 85.5 |
| **Control for long-term and seasonal trend** |  |  |  |  |  |  |  |  |  |  |  |  |  |  |  |  |
| Yes | 8 | 1.08（1.00-1.16） | 0.002 | 69.1 | 7 | 1.28（1.11-1.47） | 0.000 | 75.5 | 4 | 1.10（1.00-1.22） | 0.000 | 84.7 | 5 | 1.21（1.05-1.41） | 0.000 | 87.4 |
| No | 2 | 1.14（0.92-1.42） | 0.019 | 81.9 | 4 | 1.48（1.12-1.96） | 0.079 | 55.8 | 3 | 1.10（1.06-1.14） | 0.464 | 0.0 | 2 | 1.14（1.05-1.23） | 0.292 | 10.0 |

*RR*: Relative Risk; *CI*: Confidence Interval;
